# A formulation of pancreatic pro-enzymes provides potent anti-tumour efficacy: a pilot study focused on pancreatic and ovarian cancer

**DOI:** 10.1038/s41598-017-14571-x

**Published:** 2017-10-25

**Authors:** Macarena Perán, Elena López-Ruiz, María Ángel García, Shorena Nadaraia-Hoke, Ralf Brandt, Juan A. Marchal, Julian Kenyon

**Affiliations:** 1 Department of Health Sciences, University of Jaén, Jaén, Spain; 2 Biopathology and Regenerative Medicine Institute (IBIMER), Centre for Biomedical Research (CIBM), University of Granada, Granada, Spain; 3 Department of Oncology, University Hospital Virgen de las Nieves, Granada, Spain; 4 Biosanitary Research Institute of Granada (ibs.GRANADA), University Hospitals of Granada-University of Granada, Granada, Spain; 5 vivoPharm LLC, 1214 Research Boulevard 17036, Hummelstown PA, United States; 6 Department of Human Anatomy and Embryology, Faculty of Medicine, University of Granada, Granada, Spain; 7 The Dove Clinic for Integrated Medicine, Twyford, SO21 1RG UK

## Abstract

Proteolytic enzymes have shown efficacy in cancer therapy. We present a combination of the two pro-enzymes Trypsinogen and Chymotrypsinogen A with potent *in vitro* and *in vivo* anti-tumour efficacy. A synergetic anti-tumour effect for Trypsinogen and Chymotrypsinogen A was determined at a ratio 1:6 (named PRP) using 24 human cancer cell lines. The antiangiogenic effect of PRP was analysed by matrigel-based tube formation and by fibrous capsule formation assays. Furthermore, cell invasion and wound healing assays together with qRT-PCR determination of epithelial-to-mesenchymal transition (EMT) markers were performed on human cancer cells treated with PRP. Additionally, *in vivo* pharmacokinetic studies were implemented and the PRP’s anti-tumour efficacy was explored against orthotopic pancreatic and ovarian cancer tumours. PRP formulation was proven to inhibit *in vitro* angiogenesis, tumour growth, cancer cell migration and invasiveness; and to be an effective and well tolerated *in vivo* anti-tumour treatment. Finally, the clinical efficacy of a suppository formulation containing both pancreatic pro-enzymes in the context of a UK Pharmaceuticals Special Scheme was evaluated in advanced cancer patients. Consequently, PRP could have relevant oncological clinical applications for the treatment of advanced or metastatic pancreatic adenocarcinoma and advanced epithelial ovarian cancer.

## Introduction

Cancer is the second most common cause of death in the US. According to the American Cancer Society 42% of men and 37% of women are at risk of developing cancer during their life-time. Pancreatic cancer accounts for about 3% of all cancers in the US and about 7% of cancer deaths, while ovarian cancer ranks fifth in cancer deaths among women, accounting for more deaths than any other cancer of the female reproductive system. Since only 10% of cancer deaths are caused by the primary tumour, the therapeutic challenge is the metastatic or secondary tumour, which are generated by the cancer cell spread initiated by the loss of the adhesion molecules that hold the tumour within the original tissue^1^. In the later years, the epithelial to mesenchymal transition (EMT) process has been proposed as the initial event for tumour metastasis^2^. In fact, when a cancer cell loses its epithelial properties to acquire a mesenchymal phenotype, it acquires an increased chance to migrate to other tissues^3^. Thus, the development of novel therapeutic strategies modulating the EMT progression in cancer cells has become, in recent years, a priority with broad clinical applicability.

Although the therapeutic application of pancreatic enzymes was proposed more than 100 years ago^4^ and the combination of proteolytic enzymes has proven efficacy in cancer therapy^5,6^, most approaches are based on the combination of several proteolytic enzymes, which could increase the probability of undesired and unpredicted events. We have recently proven that the treatment of human cancer cells with Trypsinogen (T) and Chymotrypsinogen A (C) results in an enhancement of cell adhesion, an attenuation of several EMT-associated markers, and an increase in the expression of several differentiation-associated markers, suggesting the acquisition of a less malignant phenotype^7^. Furthermore, retrospective clinical cohort studies in cancer patients have demonstrated that oral enzyme therapy significantly decreased tumour- and therapy-induced side effects and symptoms, such as nausea, gastrointestinal complaints, fatigue, weight loss, and restlessness and, hence, stabilized the quality of life (QoL) of cancer patients^8–10^.

Here, we present extensive *in vitro* and *in vivo* studies to investigate the anti-tumour efficacy of a pro-enzyme formulation consisting of a combination of Trypsinogen and Chymotrypsinogen A in a synergistic ratio. First, we tested the anti-proliferative effect for the combination of T and C in 24 cancer cell lines and determined a synergistic ratio for T and C (1:6), which was defined as PRP. Second, we evaluated the *in vitro* anti-angiogenic effect of this formulation, by soft-agar tube formation assay, and *in vivo* using the AngioChamber™ assay, which is based on the normal physiological process of wound healing, to promote fibrous capsule formation around an implanted growth factor-releasing Teflon chamber^11^. Third, to analyse the anti-metastatic effect of the pro-enzyme treatment we studied the effect of PRP in cell invasion, cell migration and in the modulation of EMT related genes in pancreatic and ovarian cancer cells. Furthermore, we perform *in vivo* a PRP pharmacokinetic study and assessed the anti-tumour efficacy of PRP in murine cancer models. To this end, we treated mice orthotopically inoculated with A2780 human ovarian cancer cells or with Pan02 mouse pancreatic tumour cells with PRP. Finally, we report here clinical efficacy in 46 patients with advanced malignant disease of different origin treated with a rectal formulation of pancreatic pro-enzymes.

## Results

### Determination of optimal pro-enzyme ratio

**Figure 1:**
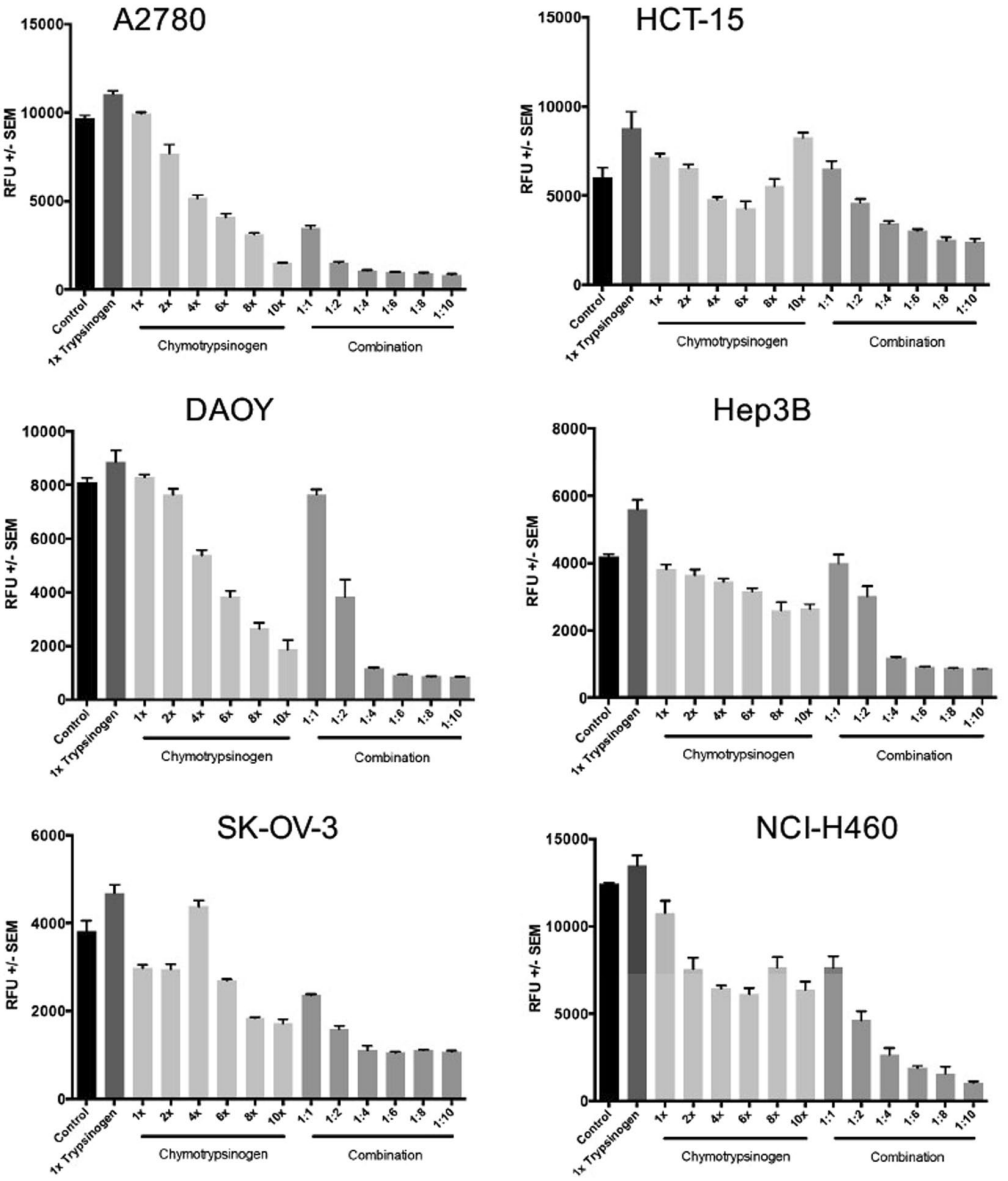
Determination of Coefficient of Drug Interaction. Combination assays of Trypsinogen and Chymotrypsinogen were performed at ratios of 1:1, 1:2, 1:4, 1:6, 1:8 and 1:10. The representative graphs show an optimal pro-enzyme synergistic ratio of the Trypsinogen to Chymotrypsinogen as 1:6.

In this study, we determined first the half-maximal inhibitory concentrations (IC_50_) of Trypsinogen and Chymotrypsinogen A as single test-article effect, in an extended panel of 24 human cancer cell lines. The IC_50_ values of Trypsinogen were the basis for the calculation of concentration ratios for the combination of Trypsinogen and Chymotrypsinogen A at 1:1, 1:2, 1:4, 1:6, 1:8 and 1:10. At these ratios the growth inhibitory properties of the combination were evaluated in 24 cancer cell lines. Based on coefficient of drug interaction (CDI) values, the combination of Trypsinogen plus Chymotrypsinogen A demonstrated greater growth inhibition at ratios of 1:4, 1:6 and 1:8 compared to the 1:1 ratio in all cell lines tested except for 786-O, G-361, BT-474 and HL-60 tumour cells (Fig. 1; Supplementary Table S4). Finally, a ratio of T:C = 1:6 of both pro-enzymes was found to be optimal and was used for the following experiments. Supplementary Table S5 summarizes IC50 values of the cell lines used for the *in vitro* experiments.

### Anti-angiogenic efficacy of pancreatic proenzyme formulation

**Figure 2:**
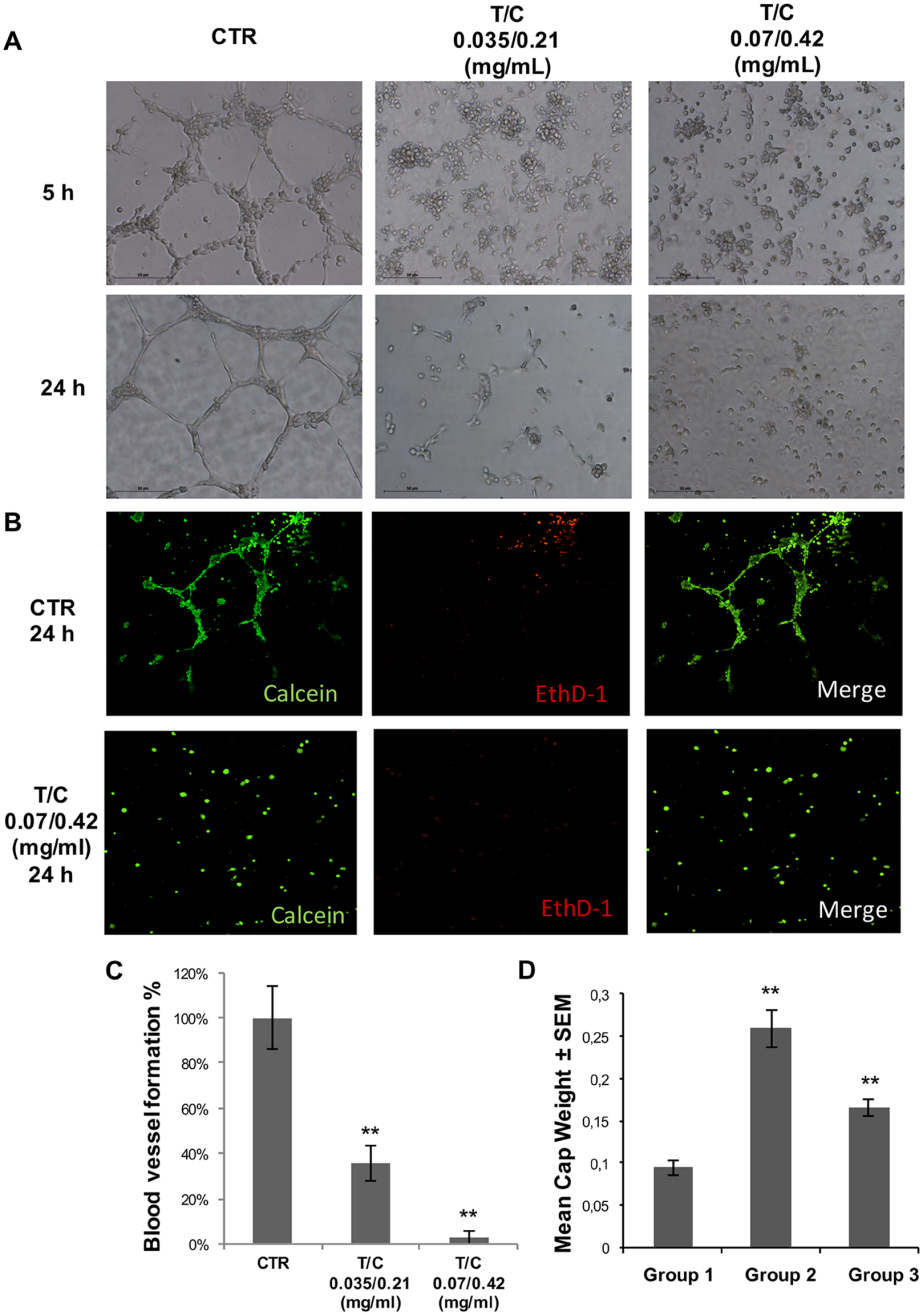
Angiogenesis tube-formation assay. (**A**) Representative light microscopy images of HUVECs in Matrigel™ assays. HUVEC cells were grown on Matrigel and the formation of capillary-like structures was assessed after 5 hrs and after 24 hrs of treatment with T/C 0.035/0.21 (mg/mL) or T/C 0.07/0.42 (mg/mL). (**B**) Cell viability assay was performed by measuring metabolically active cells stained with Calcein AM. Representative fluorescent images of HUVECs control cells (CTR) and treated cells (T/C 0.07/0.42 mg/mL) after 24 h are shown, where live cells appear green and dead cells stained with ethidium homodimer-1 (EthD-1) appear red. Original magnification 10x for all panels. (**C**) Number of capillary-like structures measured after 24 h of culture. Data from 3 independent experiments performed in duplicate are expressed as mean ± SD (**P < 0.01 vs. Control). (**D**) Mean capsule weight (g) from mice with implanted AngioChambers™ without βFGF l and treated with Vehicle Control (Group 1), containing βFGF l and treated with Vehicle Control (Group 2) and containing βFGF l and treated with T/C (0.13/0.78 mg/kg administered i.p., in a dosing volume of 10 mL/kg) (Group 3) (**P < 0.05).

To determine whether the enzyme therapy affects angiogenesis, a soft-agar tube formation assay was used. Dispersed control human umbilical vein endothelial cells (HUVEC) organized into clusters after 3 h, and began to form tube-like structures after 5 h that were clearly evident after 24 h (Fig. 2A). On the contrary, PRP treated HUVECs presented a marked reduction in the number and length of closed capillary tubes in a concentration dependent manner, with a total disappearance of the structures after treatment with T/C 0.07/0.42 mg/mL (Fig. 2A). To asses if the inhibition of the tubule-like structures formation could be due to cell death caused by PRP treatment the LIVE/DEAD Viability/Cytotoxicity Kit was used to identify viable cells. As appreciated in Fig. 2B, both control and PRP treated cells showed green staining, indicating that the inhibition of cellular cords was independent from cell viability.

Furthermore, quantification of the number of capillary-like structures at different areas of the well revealed a dramatic and significant difference between the number of structures formed by non-treated cells when compared with PRP treated cells (Fig. 2C).

The anti-angiogenic effect of PRP was additionally investigated *in vivo* using the AngioChamber™ assay, a model used to assess the efficacy of anti-angiogenic treatments by measuring fibrous capsule formation in mice. In this assay the inclusion of bFGF in the chamber supports the induction of blood vessels development and formation of a fibrous capsule. AngioChambers™ were excised from all post-mortem mice on the termination day, 24 hours following final treatment (Day 5). Figure 2D shows that fibrous capsule formation was significantly greater in the vehicle control group with bFGF captured in the chamber (Group 2, Induction Control) than in the vehicle control group without bFGF loaded into the chamber (Group 1, Baseline Control) (p < 0.05) indicating that bFGF adequately and significantly stimulated capsule formation. Treatment with PRP (Group 3) resulted in a significant reduction in angiogenesis compared to the induction control (Group 2), as indicated by the difference in capsule weight (p < 0.05) with a 57% of fibrous capsule formation inhibition. Thus, PRP inhibits fibrous capsule formation showing significant *in vivo* anti-angiogenic effects.

### Anti-invasion, anti-migration and anti-EMT effect of PRP

**Figure 3:**
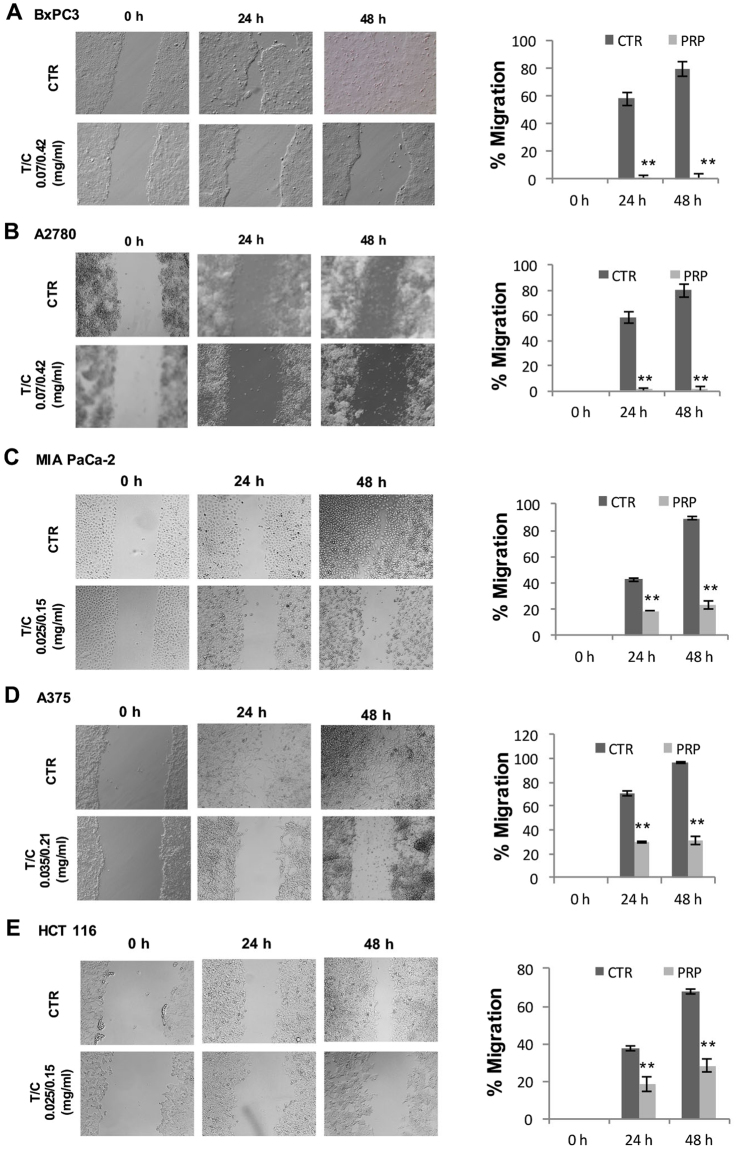
Wound healing assay to determine cell migration. Wound-healing assays were performed at 0, 24 and 48 h in BxPC3, A2780, MIA PaCa-2, A375 and HCT 116 cells in IC 50 treated cells and untreated cells used as controls. (**A**) Representative phase-contrast microscope images showing the area covered by the cells at 0, 24 and 48 h after wounding. Original magnification 10 ×. (**B**) Original magnification 10 ×. (**B**) % Cell migration was determined by the rate of cells moving towards the scratched area upon time using ImageJ™ software (^∗^P < 0.05 and ^∗∗^P < 0.01).

To analyse the *in vitro* anti-metastatic effect of the pro-enzyme treatment, we studied the effect of PRP in cell invasion, cell migration and in the modulation of EMT related genes in cancer cells. First, to evaluate the effect of PRP on cell migration, a key event in carcinogenesis, we performed a wound-healing assay on five different cancer cell lines: human pancreatic BxPC3 and Mia Paca-2, human ovarian A2780 cells, human melanoma A375 and human colon (HCT 116). Migration is defined as the directed movement of cells on a substrate such as plastic plates occurring on 2D surfaces. Here we show that non-treated cells migrated faster to close the gap of a scratch in the cell monolayer than PRP treated cells. PRP significantly reduced cell migration of pancreatic BxPC3 cells and compared with control cells even enhanced the width of the wound, a pattern of migration that was different when compared with the other cell lines and that it seemed to be cell type specific (Fig. 3A). Although the A2780 ovarian tumour cell line does not grow forming a homogeneous monolayer like BxPC3, it can be observed that PRP treatment significantly reduces the ability of the ovarian cells to migrate (Fig. 3B). Data showed significant cell migration inhibition with only a 23% of migrated cells after 48 h of treatment with PRP compared to control cells that reached 88% (Fig. 3A and B). Similar results were obtained with MIA PaCa-2, A375 and HCT 116 cells, treated cells showed values of cell migration of the order of 25–30% after 48 h of being treated with PRP, while non-treated cells gradually moved into the cell-free scratch region (Fig. 3C–E).

**Figure 4:**
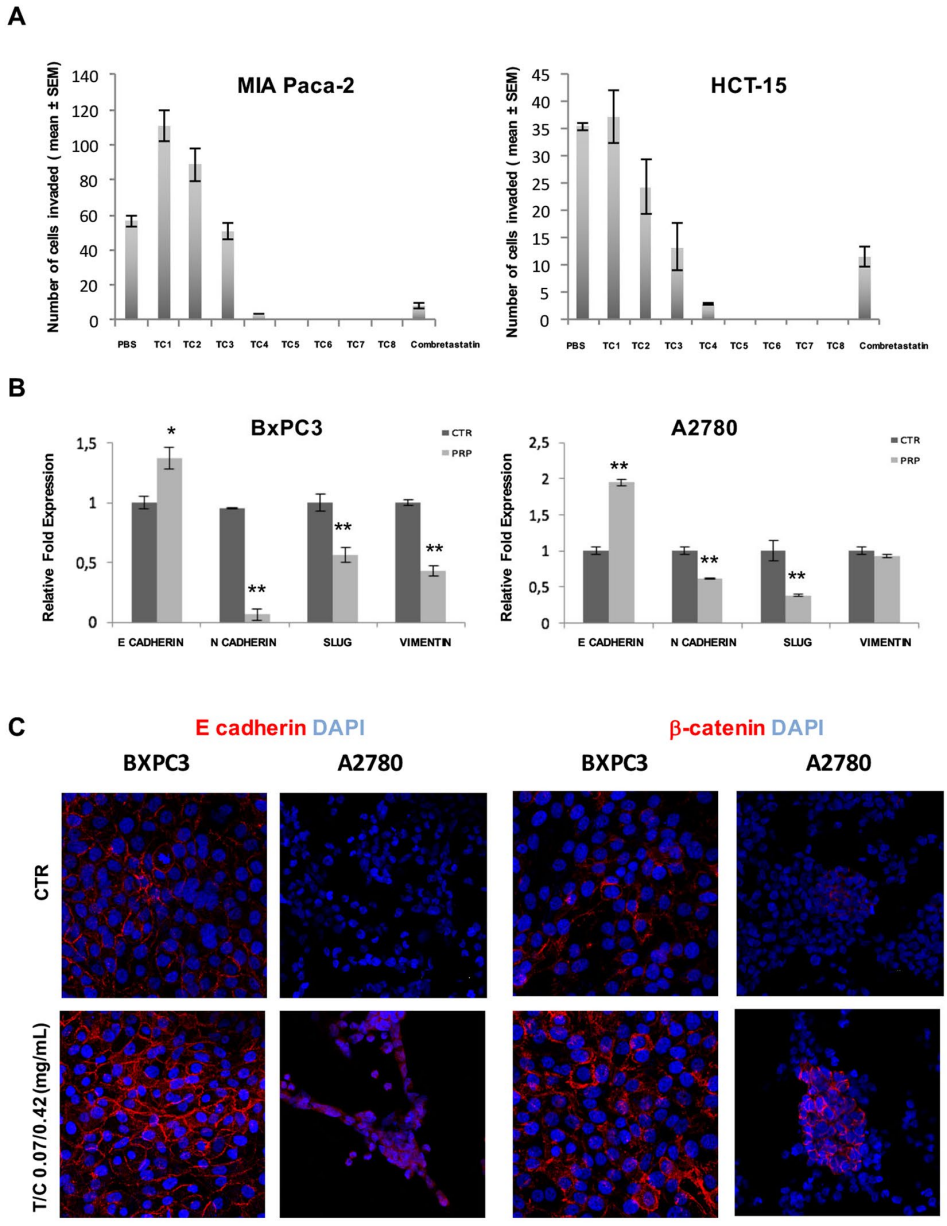
Cell invasion and EMT related genes analysis. (**A**) 3D migration assay. The graphs represent the number of cells that transmigrate through the porous membrane after exposition to increasing concentrations of PRP. PBS was used as negative control and Combretastatin as positive control. (**B**) EMT related genes expression. qRT-PCR analysis showed the increased expression of E-cadherin with concomitant significant reduction in the expression of N-cadherin, Slug and vimentin in BxPC3 cells. PRP did not significantly affect vimentin, but significantly reduced the expression of N-cadherin and Slug and increased the expression of E-cadherin in A2780 cells. (^∗^P < 0.05 and ^∗∗^P < 0.01). (**C**) Representative confocal images of E-cadherin and β-catenin expression in BxPC3 and A2780 treated and control cells. Cell surface E-cadherin and β-catenin expression was detected (in red) and nuclei were counterstained with DAPI (blue). Original magnification 40 ×.

Second, we tested the inhibitory effect of the pro-enzyme formulation on cell invasion of colon and pancreatic tumour cells. Invasion is defined as cell movement through an extracellular 3D matrix. The principle of this assay is based on two medium containing chambers separated by a porous membrane through which cells transmigrate. Here, we tested different concentrations of PRP (Supplementary Table S2) on MIA PaCa-2 pancreatic and HCT-15 colon human cancer cell lines. Interestingly, PRP showed a marked and significantly dose-dependent inhibition of invasion in both cell lines. Total inhibition of cell migration was achieved from PRP concentrations of T/C 0.015/0.093 mg/mL and so on with the other increasing concentrations tested (Fig. 4A).

To investigate whether the exposure of PRP has a potential regulation in the transcriptional machinery that drives EMT in cancer cells, expression of EMT genes were studied in BxPC3 pancreatic and A2780 ovarian human cancer cells (Fig. 4B). EMT markers in both BxPC3 and A2780 cells were affected by PRP treatment at T/C 0.07/0.42 mg/mL. Figure 4B shows that PRP treatment increased the expression of E-cadherin (0.4 fold) (*P < 0.05), while reduced the expressions of N-cadherin, Slug and vimentin (0.9, 0.5 and 0.6 fold, respectively) (*P < 0.01) in BxPC3 cells. In adittion, PRP significantly up-regulated the expression of E cadherin (0.9 fold) (**P < 0.01) and significantly down-regulated the expression of N-cadherin and Slug (0.4 and 0.6 fold, respectively) (**P < 0.01) and induced a slight, but not significant, decrease of vimentin expression in A2780 cells. Finally, immunofluorescence analysis were performed to confirm the changes in gen expression. Confocal imagines of BxPC3 and A2780 cells immunostained to detect the expression of the epithelial markers E-cadherin and β-catenin showed an increased expression of both markers in the cell membrane of PRP treated cells when compared to untreated control cells (Fig. 4C).

### PRP pharmacokinetic study

To evaluate the pharmacokinetics and organ distribution of Trypsinogen and Chymotrypsinogen A, non-tumour bearing female athymic Nude-Foxn1^nu^ mice were treated with IRDye® 800CW labelled Trypsinogen (5 mg/kg) plus unlabelled Trypsinogen (50 mg/kg) or IRDye® 800 CW labelled Chymotrypsinogen A (7 mg/kg) plus unlabelled Chymotrypsinogen A (300 mg/kg). Animals were euthanized at specified time-points post-dose and plasma along with organ homogenates was prepared, then imaged via IVIS imaging system.

**Figure 5:**
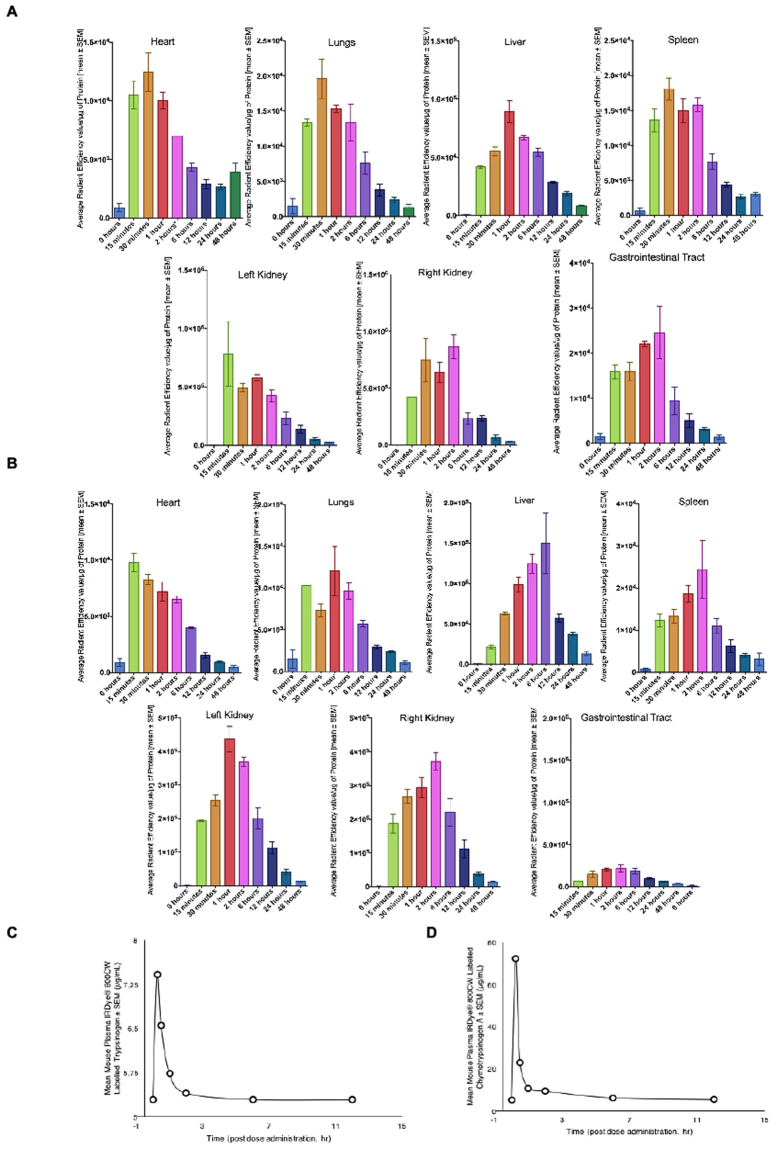
Pharmacokinetic study. Fluorescence readings in organ homogenates from mice treated with (**A**) IRDye® 800CW labelled Trypsinogen (5 mg/kg) and Trypsinogen (50 mg/kg) or (**B**) IRDye® 800CW labelled Chymotrypsinogen A (7 mg/kg) and Chymotrypsinogen A (300 mg/kg). (**C**) Levels of IRDye®800CW labelled Trypsinogen and (**D**) Chymotrypsinogen A in mouse plasma.

Fluorescence was measured in organ homogenates. Mice treated with labelled T, presented a fluorescence peak in all organs between 15 minutes and 2 h post-dose. While mice treated with labelled C showed the maximum fluorescenct emission between 15 minutes and 6 h post-dose. For both highest readings were observed in the kidneys and liver (Fig. 5A and B). Maximum levels of both IRDye®800CW labelled Trypsinogen and Chymotrypsinogen A in mouse plasma occurred at 15 minutes post-dose (7.5 and 72.2 μg/ml, respectively). Levels of both IRDye® 800CW labelled proenzymes decreased rapidly after this time (Fig. 5C and D).

### Anti-tumour efficacy of PRP in orthotopic mice models

**Figure 6:**
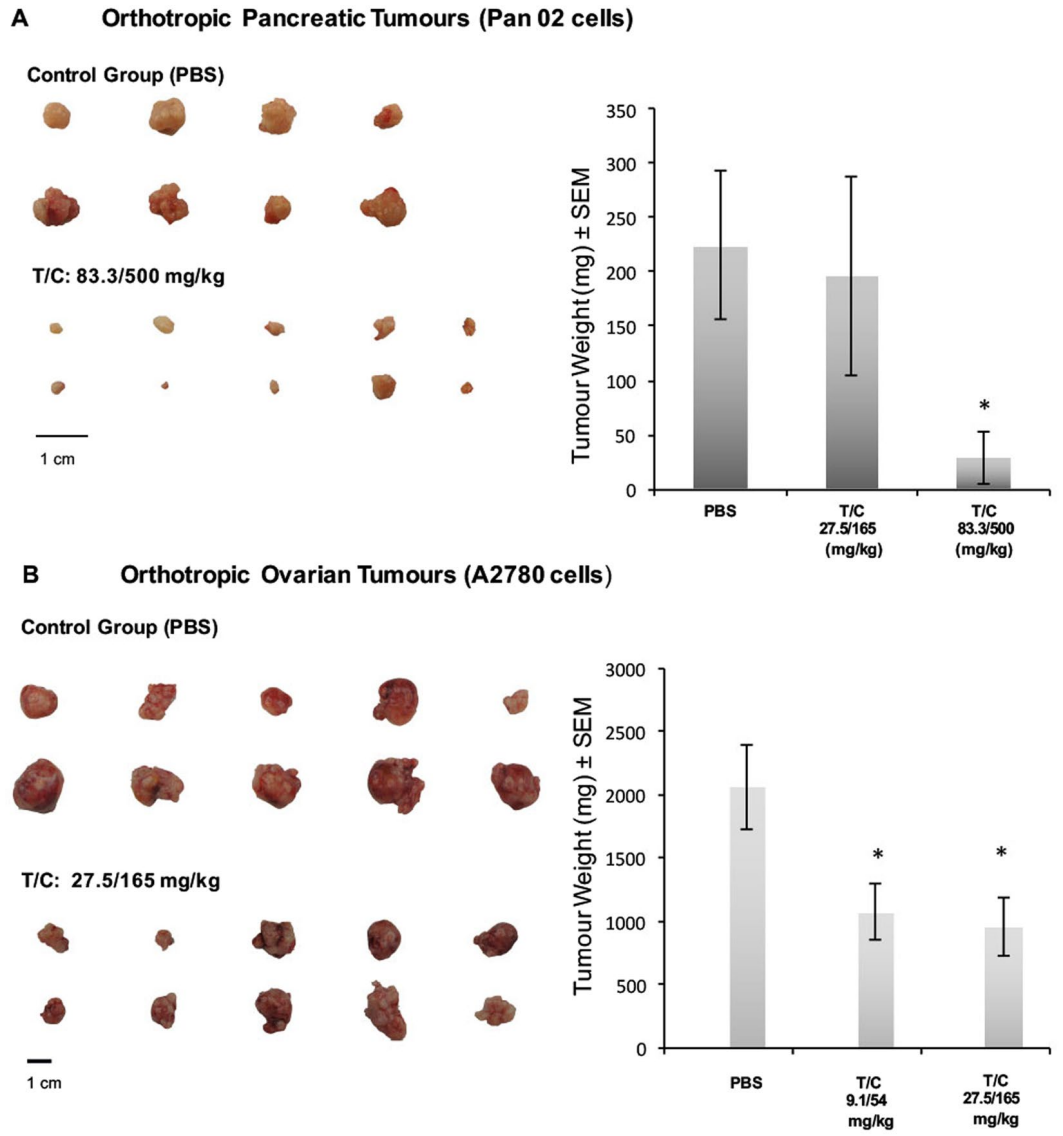
*In vivo* anti-tumour activity of PRP. (**A**) Images of excised tumours at study termination of orthotropic pancreatic tumours. Mean tumour weight at termination day after daily i.v. injection of PBS, T/C 27.5/165 (mg/kg) or T/C 83.3/500 (mg/kg) (^∗^P < 0.05). (**B**) Images of excised tumours at study termination of orthotropic ovarian tumours. Mean tumour weight at termination day after daily i.v. injection of PBS, T/C 9.1/54 (mg/kg) or T/C 27.5/165 (mg/kg) (^∗^P < 0.05).

The effect of the pro-enzyme formulation PRP at different doses on tumour weight in orthotopically implanted pancreatic and ovary tumours is graphed in Fig. 6. In the pancreatic tumour control group one animal was euthanized on day 6 due to adverse clinical signs unrelated to treatment and another animal was found dead due to unknown causes on day 23. There was significant (*P < 0.05) reduction in mean tumour weight in animals treated for 26 days with Trypsinogen/Chymotrypsinogen A at 83.3/500 mg/kg (30.2 mg; 85.9% inhibition) compared with control (PBS; 214.8 mg), but not between Trypsinogen/Chymotrypsinogen A at 27.5/165 mg/kg (196.5 mg; 8.5% inhibition) and the control (Fig. 6A).

Furthermore, ovary tumour-bearing mice (Fig. 6B) showed a significant (*P < 0.05) reduction in mean tumour weight in animals treated for 21 days with two different doses of Trypsinogen/Chymotrypsinogen A, 9.1/54 mg/kg and 27.5/165 mg/kg, compared with control (PBS). The mean weight of control group tumours was 2062.2 mg while the treated groups presented a mean tumour weight of 1074.2 mg and 957.3 respectively, ranging in a 50% tumour inhibition (52–46%).

### Overview of clinical studies

The clinical efficacy of a suppository formulation containing bovine pancreatic pro-enzymes Trypsinogen and Chymotrypsinogen A was evaluated in the context of a UK Pharmaceuticals Special Scheme. Clinical effects were studied in 46 patients with advanced metastatic cancers of different origin (prostate, breast, ovarian, pancreatic, colorectal, stomach, non-small cell lung, bowel cancer and melanoma) after treatment with a rectal formulation of both pancreatic pro-enzymes. No severe or serious adverse events related to the rectal administration were observed. Patients did not experience any haematological side effects as typically seen with classical chemotherapy regimens. No allergic reactions after rectal administration of suppositories were observed. In order to assess the therapeutic activity of rectal administration, overall survival of patients under treatment was compared to the life expectancy assigned to a patient prior to treatment start. Table 1 provides the list of patients treated with the pancreatic pro-enzyme formulation. Nineteen from 46 patients (41.3%) with advanced malignant diseases, most of them suffering from metastases, had a survival time significantly longer than the expected life span, in fact, for the whole set of cancer types, mean survival (9.0 months) was significantly higher than mean life expectation (5.6 months).

**Table 1:** Overview of clinical studies. Patients who met prognosis of life expectation (*). For the whole set of cancer types, mean survival (9.0 months) was statistically significant higher than mean life expectation (5.6 months). One way ANOVA (α = 0.05, P < 0.05).

Cancer Type	Life Expectation (months)	Survival ** (months)
Pancreatic carcinoma (n = 4)	2	8
	4	*
	<3	7
	<3	4
Ovarian Cancer (n = 7)	4	11
	6	12
	6	11
	<12	38
	<1	1
	4	*
	3	*
Breast Cancer (n = 6)	6	9
	6	*
	2	*
	12	*
	<12	*
	12	*
Colon Rectal Cancer (n = 5)	6	*
	6	*
	12	*
	6	40
	12	*
Gastric Cancer (n = 2)	2	8
	<3	7
Prostate Cancer (n = 8)	4	*
	1	5
	4	*
	<12	*
	12	14
	12	*
	12	*
	12	*
Non-Hodgkin Lymphoma (n = 1)	2	9
Mesothelioma (n = 1)	3	9
Melanoma (n = 2)	6	*
	<3	4
Neuro-endocrine Tumour (n = 1)	10	24
Bladder (n = 2)	<3	*
	12	*
NSCLS (n = 2)	3	5
	6	*
Bowel (n = 2)	<12	*
	<3	3
Small Cell Carcinoma (n = 1)	<12	*
Renal Cancer (n = 1)	<3	*
Abdomen unknown primary (n = 1)	<12	*

Although the number of patients per cancer indication is naturally quite low, 3 out of 8 patients with prostate cancer and 5 out of 11 patients with gastrointestinal cancers appear to particularly benefit from the treatment with the pro-enzyme suppositories.

## Discussion

The presented data have been relevant to adopt a new strategy in the formulation of a preparation composed of pancreatic pro-enzymes (PRP) that proved to reduce the main characteristics of cancer spread. In this context, angiogenesis, the ability to form new blood vessels, represents a critical step in tumour development through which tumours establish their own blood supply^12^. Therefore, inhibition of angiogenesis will block one of the fundamental requirements for tumour growth as well as the spreading of tumour metastases to secondary tumour sites. We investigated the *in vitro* anti-angiogenic activity of PRP using the tube formation assay and the soft-agar tube formation assay and *in vivo* with the AngioChambers™ assay. Results showed that PRP inhibits the formation of capillary-like structures of HUVECs and suppress the fibrous capsule formation driven by the bFGF captured within the subcutaneously implanted AngioChambers^TM^ and subsequently by stimulation of endogenous VEGF. Angiogenic factors such as bFGF and VEGF, secreted by many cells including macrophages and cancer cells, have been shown to play an important role in progression of pancreatic carcinoma^13^ and other tumour types^14–16^. Indeed, bFGF stimulates all major steps in the angiogenesis cascade as is known to be involved in proliferation, migration and differentiation of endothelial cells^17^.

Another key factor in cancer progression is the loss of cell-cell adhesion, which correlates with an increased capacity of cell migration and the acquisition of invasive properties by the malignant cells, which undergo a morphological change adapting a spindle like appearance^18^. In addition, an *in vitro* wound healing assay revealed that the migration capacity of ovarian pancreatic, melanoma and colon cancer cancer cells was suppressed after incubation with PRP. Furthermore, the inhibition of cell invasion potential most likely bears significant potential to inhibit *in vivo* metastasis.

To understand the possible molecular mechanisms that inhibit migration and invasion of PRP treated cells, qRT-PCR analysis was performed to study the expression of EMT related genes, a process in which epithelial cells drop their characteristics and undergo transdifferentiation into motile mesenchymal cells. Evidence suggests that EMT is associated with metastasis and with the acquisition of chemoresistant properties in many types of cancers^19^ including ovarian^20^ and pancreatic cancer^21^. Indeed, previous studies have reported that during ovarian cancer progression, EMT plays an important role in increasing cell invasion, which lead to the poor outcome of ovarian cancer patients^22,23^. In addition, studies have found that EMT contributes to resistance to chemotherapeutic drugs in pancreatic cancer^24^ and several EMT markers have also been recognized in pancreatic cancer^25,26^. In this study, we demonstrate that PRP treatment promoted the gene expression of the epithelial marker E-cadherin and decreased expression of both mesenchymal markers, N-cadherin and vimentin. A key feature of EMT is the switch from epithelial markers, such as E-cadherin and cytokeratin to mesenchymal markers such as N-cadherin, vitronectin 75 or vimentin^27,28^. Here we show that ovarian and pancreatic cancer cell lines showed a significant decrease in N-cadherin expression in response to PRP treatment. This is a highly relevant observation, since N-cadherin up-regulation is associated with an increased migration and invasion potential of ovarian and pancreatic cancer cells^29–31^. In addition, several studies have pointed to the importance of N-cadherin signalling in pancreatic cancer progression^32,33^. It has been reported that N-cadherin knockdown in BxPC3 cells leads to an increased expression of E-cadherin and also resulted in a significant reduction in the motility of BxPC3 cell line, suggesting that N-cadherin is a key mediator of the EMT process in pancreatic cells^28^.

In addition, protein expression of the epithelial markers E-cadherin and β-catenin was enhanced after PRP treatment, and appeared restricted to the cell membrane when analysing by immunofluorescence. E-cadherin is an important signalling molecule in EMT in part via its interaction with β-catenin^18^, in fact, reduction in E-cadherin and β-catenin expression has been associated with a diminution of cell adhesion^7^. Moreover, β-catenin translocation to the nucleus and decrease expression of E-cadherin are observed in various human malignancies associated with metastasis^20^.

Furthermore, one of the genes strongly inhibited by PRP treatment in both BxPC3 and A2780 cell lines was Slug, a transcription factor that has been shown to repress E-cadherin and regulate other EMT markers. Moreover, it has been reported that Slug is a strong inductor of VEGF and it shows potent pro-angiogenic effects^34^. Also, Slug is closely associated with tumour metastasis and angiogenesis in ovarian cancer^35^ and with the regulation of the expression of the actin bundling protein fascin, often highly elevated in malignant tumours, and recently suggested as a marker or therapeutic target for pancreatic cancer^36^. Therefore, our data suggest that PRP reverts EMT in cancer cells.

Intra venous injection of the pro-enzymes resulted in maximum detectable levels in plasma at 15–30 min that decline in the first hour and were still present in low levels after three hours post-dose. These observations are similar to time-concentration profiles found in previous pharmacokinetic studies with potential therapeutic agents for human cancers, including pancreas^37,38^ and also in current chemotherapeutics used in the clinic such as gemcitabine^39^. Moreover, examination of the tissue distribution demonstrated that proenzymes perfused into organs, including the heart, liver, spleen, lung, kidney and entire gastrointestinal tract which indicated a wide tissue distribution of the pro-enzymes^40^.

We further investigated orthotopic tumour models to test the anti-tumour efficacy of PRP using human tumour cells (A2780) or murine tumour cells (Pan 02). Clinical observations have suggested that the organ environment can influence the response of tumours to chemotherapy. Likewise, orthotopic implantation of human tumour cells into nude mice provides advantages for the study of tumour growth and metastasis^41^. In adition, Panc 02, has proven to be a suitable syngeneic pancreatic cancer cell line for developing orthotopic tumours in immunocompetent murine model that closely mimics human pancreatic cancer^42^. One of the reported advantages of orthotopic models is that targeting processes involved in local invasion (e.g., angiogenesis) can be undertaken at a more clinically relevant site. Therefore, the anti-tumour efficacy of daily treatment with Trypsinogen and Chymotrypsinogen A, administered in combination as a single i.v. injection at two doses was assessed against human ovarian and pancreatic cancer cells, orthotopically inoculated in female athymic nude-Foxn1^nu^/^nu^ mice or female C57BL/6 mice, respectively. While the two doses tested were effective against the orthotopic ovarian tumours, only the higher dose tested was effective in the pancreatic tumours. Of note, the poor response of pancreatic tumours to available therapies is well known^43^.

PRP not only showed to dicreassed tumour size but also was proven to be a non-toxic anti-cancer agent because no adverse clinical events related with the treatment were found. Taken together, the presented pro-enzyme formulation (PRP) significantly inhibits human pancreatic and ovarian tumours, both well vascularized and relatively fast growing tumour entities. For both indications, pancreatic and ovarian cancer, available clinical therapy is limited and, thus, present attractive indications for further clinical development of PRP.

Finally, in the context of a UK Pharmaceuticals Special Scheme we evaluated the clinical efficacy of a suppository formulation, containing Trypsinogen, Chymotrypsinogen A and traces of α-amylase, in patients with several different malignancies. α-Amylase was included because it was part of crude pancreatic extracts that have been used in the early days of enzyme therapy, but after evaluating the synergistic interaction between Trypsinogen, Chymotrypsinogen and α-amylase it was concluded that α-amylase did not contribute to the anti-tumor activity of the formulation.

By rectal administration, the treatment was well tolerated by patients and has the advantage that the formulation is absorbed by the rectum’s blood vessels into the circulation, avoiding digestion of the enzymes in the duodenum. Therefore, a rectal formulation leads to improved efficacy over current oral enzyme therapies and would reduce the high doses historically used for oral applications^44^. There is some controversy in the literature as to the amount of intact pro-enzymes that actually reaches the blood stream when administered orally, but also rectally. While some authors contend that only a small fraction (0.002%)^45^ or even none^46^ is absorbed, an alternative theory suggests that these enzymes are acid stable and are absorbed through the gastrointestinal mucosa into the blood stream as part of an enteropancreatic recycling process^47^. Moreover, clinical efficacy of rectal pancreatic pro-enzymes, as judged by extended survival time in comparison to predicted life expectancy at the commencement of treatment, was evident in a number of patients.

Summarizing, PRP proves to be an *in vivo* effective and non-toxic anti-tumour treatment, able to inhibit angiogenesis and tumour growth, cancer cell migration and invasiveness. Furtheremore, a suppository formulation containing both pancreatic pro-enzymes showed to increase the life expectancy of advanced cancer patients. Consequently, PRP could have relevant oncological clinical applications for the treatment of solid tumours like advanced pancreatic adenocarcinoma and advanced epithelial ovarian cancer.

## Materials and Methods

### Cell culture

Cancer cell lines were sourced from the American Type Culture Collection (ATCC) (Rockville, MD, USA). Supplementary Table S1 shows the cell lines used and the culture growth conditions for each of them. All cells were cultured at 37 °C in a humidified cell culture incubator supplied with 95% air/5% C0_2_.

### Animal care compliance

Procedures involving the care and use of animals in the study were reviewed and approved by the Pennsylvania State College of Medicine Institutional Animal Care and Use Committee (IACUC) prior to conduct. During the study, the care and use of animals were conducted in accordance with the principles outlined in the Guide for the Care and Use of Laboratory Animals, 8^th^ Edition, 2010 (National Research Council).

### *In vitro* cytotoxicity assays and coefficient of drug interaction determinations

Trypsinogen (T) and Chymotrypsinogen A (C) were dissolved in PBS, stock solutions were sterilised by filtration (0.22 µm) and for each pro-enzyme formulation stocks were mixed into the appropriate combinations. To determine the *in vitro* cytotoxicity (half maximal inhibitory concentrations, IC_50_) and the CDI, 96 well plates were seeded for each cell line, pro-enzyme combinations were added to cells 24 hours post-seeding and tested in triplicates for each cell line. At 72 hours post addition, the CellTiter-Blue® Assay was carried out on all plates following manufacturer’s instructions. Briefly, 10 µL of CellTiter-Blue® was added to each well and incubated with cells for up to 4 hours. Fluorescence was measured using a Spectramax Gemini XPS Fluorometer. All data were recorded and entered into Microsoft® Excel spread sheets for interpretation. Data collected from CellTiter-Blue® assays were plotted as dose response curves for IC_50_ determination, and as isobolograms to assess CDI.

### Matrigel tube formation assay

Human umbilical vein derived endothelial cells (HUVECs) were cultured in complete EGM-2 growth medium (Lonza, Basel, Switzerland) under standard cell culture conditions of 37 °C and 5% CO_2_. Cells were used between passages 4 and 5. Multiwell dishes (12-well plates) were coated with 300 μL Matrigel (Collaborative; BD PharMingen, San Diego, CA) at 4 °C and were incubated for 30 minutes at 37 °C. HUVECs (1.2 × 10^5^ cells/well) were added to the Matrigel-coated wells in complete culture medium. Pro-enzyme formulation was added after 20 min of seeding on Matrigel at 2 concentrations, T/C: 0.035/0.21 mg/mL and T/C: 0.07/0.42 mg/mL. After 5 and 24 hours of incubation, cells were photographed under phase-contrast microscopy (Leica DM 5500B, Solms, Germany). The ability of cells to form capillaries was examined and the number of capillary-like structures was measured. Each cord portion between the ramifications was considered as one capillary unit. Mean ± standard deviation (SD) values were obtained by evaluating four representative areas of each culture from three independent experiments.

Cell viability staining was performed by the LIVE/DEAD Viability/Cytotoxicity Kit (Molecular Probes), according to the manufacturer’s instructions. In brief, HUVECs (3 × 105 cells/well) were culture in Matrigel-coated wells in complete culture medium. Pro-enzyme formulation was added after 20 min of seeding on Matrigel at T/C: 0.07/0.42 mg/mL and the cell viability was tested after 24 hours of culture with Calcein AM, a probe for intracellular esterase activity, that labelled live cells, and ethidium homodimer-1, a probe for loss of plasma membrane integrity, that labelled dead cells. Cells treated with 70% methanol served as controls for dead cells (data not shown). Images were taken by confocal microscopy (Nikon Eclipse Ti-E A1, USA) and analyzed using NIS-Elements software.

### AngioChamber™ implantation and treatment

Porous Teflon tissue chambers (AngioChambers™)^48,49^ were filled under sterile conditions with 0.8% agarose containing 20 IU/mL heparin, with or without 4 µg/mL basic fibroblast growth factor (bFGF). 30 female FvB mice each received a subcutaneously implanted AngioChamber™, with or without basic fibroblast growth factor (bFGF). 30 mice were randomised by body weight into 3 groups of 10 mice each (Group 1, 2 and 3) Ten mice were implanted with chambers without bFGF (Group 1) and 20 mice, implanted with chambers containing bFGF (Group 2 and 3). Control groups (Group 1 and 2) received an oral dose of NMP:PEG300 as a vehicle control and treated mice (Group 3) received daily intraperitoneal injection (i.p.) with T/C: 0.13/0.78 mg/mL in a dosing volume of 10 mL/kg. Animals were treated 2 hours after the mice recovered from surgery to implant the AngioChamber™ on Study Day 0. AngioChambers™ were excised from all mice post-mortem on the termination day, 24 hours following final treatment (Day 5). The vascularised fibrous capsule that had formed around each chamber was carefully removed and the wet weight recorded immediately. Angiogenesis inhibition by pro-enzymes (%) was calculated using

1 $$ \% \,{\rm{Inhibition}}=[(A-B)/(A-C)\times {\rm{100}}];$$

where A is the mean capsule weight from mice with implanted AngioChambers™ containing growth factor and treated with Vehicle Control (Group 2), B is the mean capsule weight from mice with implanted AngioChambers™ containing growth factor and treated with proenzyme (Group 3), and C is the mean capsule weight from mice with implanted AngioChambers™ without growth factor and treated with Vehicle Control (Group 1).

### *In vitro* wound-healing assay

Pancreatic (BxPC3 and Mia Paca-2), ovarian (A2780), melanoma (A375) and colon (HCT 116) cancer cells were seeded in 6-well plates and grown to 80% confluence. Wounds were created by scraping monolayer cells with a 200 μl pipette tip, and non-adherent cells were washed off with medium. BxPC3 and A2780 cancer cells were treated with T/C: 0.07/0.42, mg/mL, Mia Paca-2 and HCT 116 cancer cells were treated with T/C: 0.025/0.15 mg/mL, and A375 cancer cells were treated with T/C: 0.035/0.21 mg/mL,. At 0, 24 and 48 h after the creation of wounds, treated and control non-treated cells were observed and migration images were captured with a 10x objective in a phase-contrast microscopy. Cell migration was determined by the rate of cells moving towards the scratched area. ImageJ™ software was used to quantify the scratched area. All experiments were plated in triplicate wells and were carried out at least three times.

### Matrigel cell invasion assay

Cell culture inserts (8 µm pore size) were washed twice with serum-free RPMI or DMEM medium and placed into the wells of a 24-well cell culture plate. Using cold pipette tips, 40 µL of Matrigel diluted 1:10 in RPMI medium were added to each insert, rocking the plate gently to evenly distribute the Matrigel across the surface of the membrane. Matrigel was allowed to solidify by incubating overnight at 37 °C. The following day, 7.5 × 10^4^ MIA PaCa-2 cells in 200 µL of DMEM medium supplemented with 0.1% BSA, or 7.5 × 10^4^ HCT-15 cells in 200 µL of RPMI medium supplemented with 0.1% BSA, were added to the upper chamber of each insert. Cell suspensions contained pro-enzyme combination at eight concentrations (Supplementary Table S2) or PBS (untreated control). Combretastatin A4 was used as a control of the inhibition of cell migration^50^ at a final concentration of 250 nM. Each sample was analysed in duplicate. Conditioned medium from U87-MG cells (500 µL) was used as the chemoattractant in the lower chamber^51,52^. The cells were allowed to migrate through the extracellular matrix for 24 hours at 37 °C. The non-migrating cells were removed from the upper chamber with a cotton tip. Cells on the bottom side of the membrane were fixed in 70% ethanol for 15 minutes and stained with 10% filtered Giemsa stain for 1 hour at room temperature. Migration was quantified by counting cells in three fields of view at 40x magnification using an inverted light microscope (Olympus).

### RNA isolation and quantitative real time RT-PCR assay

A2780 and BxPC3 were grown in 6 well plates and treated twice with PRP (T/C 0.07/0.42 mg/mL) on day 2 and on day 4. On day 5, total cellular RNA was isolated using TriReagent (Sigma) and reverse transcribed using the Reverse Transcription System kit (Promega). Real-time PCR was performed using the SYBR-Green PCR master mix (Promega) according to the manufacturer’s recommendations. PCR reactions were performed as follows: an initial denaturation at 95 °C for 2 min, 40 cycles of 95 °C for 5 s and 60 °C for 30 s, and final cycle of dissociation of 60–95 °C. The gene expression levels were normalized to corresponding GAPDH values and are shown as fold change relative to the value of the control sample. Untreated cells were used as a control. All the samples were done in triplicate for each gene. Primers used are shown in Supplementary Table S3.

### Immunohistochemical analysis

A2780 and BxPC3 were grown on glass coverslips and treated twice with PRP (T/C 0.07/0.42 mg/mL) on day 2 and on day 4. For immunofluorescence staining, samples were fixed with 4% paraformaldehyde (PFA) in PBS for 20 min at RT. Cells were blocked for 1 hour at room temperature (RT) with 5% BSA, 5% foetal bovine serum in PBS and then incubated with E-Cadherin (SC-21791, St. Cruz) and β-catenin (SC-7963, St. Cruz) antibodies overnight at 4 °C. The next day, samples were washed thrice with PBS and incubated with appropriate secondary antibodies (Santa Cruz) for 1 hour at RT, washed thrice with PBS and then mounted with mounting medium and DAPI. Images were taken by confocal microscopy (Nikon Eclipse Ti-E A1, USA) and analyzed using NIS-Elements software.

### Pharmacokinetic study

Conjugation of Trypsinogen and Chymotrypsinogen A to IRDye® 800CW label was done following manufacturer’s protocol (LI-COR biotechnology). Briefly, Trypsinogen and Chymotrypsinogen A were dissolved to 1 mg/ml (w/v) solution and the 50 mM potassium phosphate was used to adjust pH to 8.5. The protein and IRDye® 800CW dye mixture was incubated for 2 hrs at ambient temperature. After column filtration to remove unconjugated dye and exchange buffer to phosphate buffered saline (PBS), pH 7.2, the final protein concentration and degree of labelling (dye to pro-enzyme ratio) was determined by measuring the absorption at 280 and 780 nm. The labelled product was stored at 4 °C until used.

Biodistribution studies were performed on non-tumour bearing female athymic Nude-Foxn1nu mice (n = 3 per time point). Combination of IRDye® 800CW labelled and unlabelled pro-enzymes were administered (intravenously) to each mouse at (IRDye® 800CW labelled Trypsinogen (5 mg/kg) with unlabelled Trypsinogen (50 mg/kg) or IRDye® 800 CW labelled Chymotrypsinogen A (7 mg/kg) with unlabelled Chymotrypsinogen A (300 mg/kg). Mice were euthanized at pre-determined time-points (0 min, 15 min, 30 min, 1hr, 2 hr, 6 hr, 12 hr, 24 hr and 48 hr post-injection) and the pertinent organs (blood processed to plasma, liver, lung, heart, spleen, entire gastrointestinal tract and both kidneys) were excised, homogenized, and imaged via IVIS Lumina XR from Perkin Elmer. IRDye® 800CW labelled protein concentrations (Fluorescence intensity/mg of protein) were analysed at each time point for plasma and organ homogenate samples.

### *In vivo* orthotopic tumour models

40 female athymic Nude-Foxn1^nu^ mice were orthotopically inoculated (right ovary) with 1 × 10^6^ A2780 human ovarian cancer cells; and 40 female C57BL/6 mice were orthotopically inoculated (pancreas tail) with 1 × 10^6^ Pan02 mouse pancreatic tumour cells. 10 animals in each group remained untreated for tumour take-rate assessment. After 7 days post-inoculation (Day 0), animals were randomised into 3 sets of 10, (ovarian tumour group) and 3 sets of 10 (pancreas tumour group).

A single tail vein injection in a dosing volume of 10 mL/kg was administered once daily to each animal. The volume of dosing solution administered to each animal was calculated and adjusted based on individual body weight measured immediately prior to dosing. Treatments commenced on Day 0 and were administered for 21 consecutive days. Orthotropic ovarian tumour animals were injected with PBS (Group 1) or T/C at doses of 27.5/165 mg/kg (Group 2) and 83.3/500 mg/kg (Group 3). Orthotropic pancreas tumour animals were injected with PBS (Group 1) or T/C at doses of 9.1/54 mg/kg (Group 2), and 27.5/165 mg/kg (Groups 3). Upon termination, the ovary and the pancreas were excised from all animals with tumour intact. The tumour was isolated, weighed and then photographed. Percentage change in mean tumour weight for treated groups relative to the control group was calculated using:

2 $${\rm{Percentage}}\,{\rm{change}}=((\mathrm{Mean}\,{\rm{Control}}-{\rm{Mean}}\,\mathrm{Treatment})/\mathrm{Mean}\,{\rm{Control}})\times {\rm{100}}{\rm{.}}$$

### “Specials” licence treatment with pancreatic proenzyme formulation

46 patients with advanced malignant disease of different origin were treated daily with a rectal formulation containing 8.92 mg of each of the two pancreatic pro-enzymes and 1.78 mg α-Amylase (A) per suppository. The study was carried out under a UK “Specials” License at the Dove Clinic, Hampshire, UK for periods up to 14 months. Specials License regulation provides a legal framework for the use of a medicinal product without marketing authorisation or product license by a treating physician in order to fulfil the special needs of a patient for whom he/she is directly responsible. Treatment under a Specials License can thus not be regarded as a formal clinical study. Informed concern was obtained from all patients prior to treatment. The data reported were generated from patient notes held by the Dove Clinic.

With no formal inclusion and exclusion criteria the patient group of this compassionate use programme was highly heterogeneous. Most patients had been diagnosed with metastatic cancers of various origins, including prostate (n = 8), breast (n = 6), ovarian (n = 7), pancreatic (n = 4), colo-rectal (n = 5), gastric cancer (n = 2), melanoma (n = 2), blander cancer (n = 2), bowel cancer (n = 2), non-small cell lung (n = 2) and neuro endocrine tumour, small cell carcinoma, non-Hodgkin lymphoma, mesothelioma, neuro-endocrine tumour, small cell carcinoma, renal cancer and as well as abdomen unknown primary (all n = 1). In order to assess the treatment benefit, survival under treatment was compared to the life expectancy individually assigned prior to treatment start. This life expectancy was determined for each patient considering i) the date of the clinical diagnosis, ii) the age of the patient and comorbidities, iii) previous treatments and how they were tolerated, iv) standard blood and relevant biomarkers, v) historic scans, as no further scans were offered during the treatment period, and vi) general clinical status of the patient. These data together with published information regarding the specific tumor were taken into consideration when the expected survival for individual pancreatic patients was estimated. Patient’s life expectancy, according to oncologist’s predictions, ranged between a few days and about 12 months.

### Statistical calculations

Significant difference were tested by One Way ANOVA. Assumptions of analysis of variance (homocedesticity and normality) were tested and assured by using transformed data sets when necessary. Significance was accepted at P < 0.05 in all cases.

### Data availability statement

The datasets generated during and/or analysed during the current study are available from the corresponding author on reasonable request.

## Electronic supplementary material

Supplementary Information
