# Supplementary material for: A formulation of pancreatic pro-enzymes provides potent anti-tumour efficacy: a pilot study focused on pancreatic and ovarian cancer
Source: Sci Rep. 2017 Oct 25;7:13998. doi: 10.1038/s41598-017-14571-x (PMC5656641; doi:10.1038/s41598-017-14571-x)
Supplement: Supplementary file 1 — Supplementary Information [file 41598_2017_14571_MOESM1_ESM.pdf]

A formulation of pancreatic pro-enzymes provides potent anti-tumour efficacy: a pilot study focused on pancreatic and ovarian cancer

Macarena Perán, Elena López-Ruiz, María Ángel García, Shorena Nadaraia-Hoke, Ralf Brandt, Juan A. Marchal & Julian Kenyon

Table S1. Cell lines and culture conditions

Cell Line	Tumour Type	Culture conditions
ACHN	Renal Cell Adenocarcinoma	EMEM + 10% FCS+ 1% GlutaMax + 1% Pen/Strep
HCT-15	Colorectal Adenocarcinoma	RPMI+ 10% FCS+ 1% GlutaMax + 1% Pen/Strep
HCT-116	Colorectal Adenocarcinoma	RPMI+ 10% FCS+ 1% GlutaMax + 1% Pen/Strep
HT-1080	Fibrosarcoma	EMEM (EBSS) + 1% NEAA + 10% FCS+ 1% GlutaMax +1% Pen/Strep
HT-29	Colorectal Adenocarcinoma	RPMI+ 10% FCS+ 1% GlutaMax + 1% Pen/Strep
Hep3B	Hepatocellular Carcinoma	EMEM (EBSS) + 1% NEAA + 10% FCS+ 1% GlutaMax +1% Pen/Strep
MIAPaCa-2	Carcinoma	DMEM+ 10%FCS +1% GlutaMax + 1% Pen/ Strep+ 1% HEPES+ 1% NaPy
DAOY	Desmoplastic Cerebellar Medulloblastoma	MEM+ 10%FCS+1% GlutaMax+ 1% Pen/Strep
A2780	Ovary adenocarcinoma	RPMI+ 10% FCS+ 1% GlutaMax +1% Pen/Strep
G361	Malignant melanoma	McCoy′s 5a + 10% FCS+ 1% GlutaMax +1% Pen/Strep

SK-OV-3	Adenocarcinoma , ovary	RPMI+ 10% FCS+ 1% GlutaMax + 1% Pen/Strep
BT-474	Breast carcinoma	RPMI+ 10% FCS+ 1% GlutaMax + 1% Pen/Strep +1% NaPy
MDA -MB-231	Breast adenocarcinoma	DMEM+ 10%FCS+ 1% GlutaMax+ 1% Pen/Strep
MCF-7	Breast adenocarcinoma	EMEM (EBSS) + 1% (NEAA) + 10% FCS+ 1% GlutaMax +1% Pen/Strep
DU145	Prostate carcinoma	DMEM+ 10%FCS + 1% Pen/Strep
MES-SA	Uterine Sarcoma	McCoy′s 5a + 10% FBS + 1% GlutaMax + 1% Pen/Strep
Huh-7	Hepatocarcinoma	DMEM+ 10%FCS + 1% Pen/Strep
NCI-H460	Pleural effusion	RPMI+ 10% FCS+ 1% GlutaMax + 1% Pen/Strep
SNB-19	Glioblastoma	DMEM+ 10%FCS + 1% Pen/Strep
PC3	Prostate carcinoma	Coons Modified Ham's F12 + 7% FBS + 1% GlutaMax +1% Pen/Strep
HL60	Promyelocytic leukaemia	RPMI+ 10% FCS+ 1% GlutaMax + 1% Pen/Strep
U87-MG	Glioblastoma	MEM+ 10% FCS+ 1% GlutaMax + 1% Pen/Strep +1% NaPy
786-O	Renal adenocarcinoma	RPMI+ 10% FCS+ 1% GlutaMax + 1% Pen/Strep
C8161.9	Malignant melanoma	RPMI+ 10% FCS+ 1% GlutaMax + 1% Pen/Strep
Pan02	Mouse pancreatic tumour cells	RPMI+ 10% FCS+ 1% GlutaMax + 1% Pen/Strep
BxPC3	Pancreatic tumour cells	RPMI+ 10% FCS+ 1% Pen/Strep
A375	Malignant melanoma	DMEM+ 10%FCS + 1% Pen/Strep

Table S2. Concentrations of pancreatic pro-enzyme used for invasion assay

	TC1	TC2	TC3	TC4	TC5	TC6	TC7	TC8
Trypsinogen (μg/mL)	1.95	3.90	7.81	15.62	31.25	62.5	125	250
Chymotrypsinogen (μg/mL)	11.71	23.43	46.87	93.75	187.5	375	750	1500

Table S3. Primers used in the Real Time-PCR analysis.

**Gene**	**Forward**	**Reverse**
E-cadherin	AGAACGCATTGCCACATACACT	TCTGATCGGTTACCGTGATCAA
N-Cadherin	TGAGCCTGAAGCCAACCTTA	AGGTCCCCTGGAGTTTTCTG
Vimentin	GAACCTGAGGGAAACTAATC	GAAAGGCACTTGAAAGCT
Slug	TGGTTGCTTCAAGGACACAT	GTTGCAGTGAGGGCAAGAA

Table S4. CDI, Coefficient of drug interaction, CD1 <1, = 1 or >1 indicates that the drugs are synergistic, additive or antagonistic respectively.

	**Trypsinogen:Chymotrypsinogen**					
	1:1	1:2	1:4	1:6	1:8	1:10
**CELL LINES**	**CDI**					
786-O	0.16	0.15	0.16	0.21	0.24	0.30
C8161.9	0.70	0.42	0.31	0.31	0.19	0.19
HCT-116	0.76	0.51	0.45	0.36	0.16	0.21
HCT-15	0.62	0.48	0.49	0.49	0.31	0.20
HT-29	2.44	0.66	0.55	0.46	0.7	0.81
MES-SA	0.80	0.42	0.23	0.32	0.48	0.59
MIAPaCa-2	0.75	0.45	0.33	0.21	0.25	0.33
MDA-MB-231	1.29	0.89	0.75	0.80	0.86	0.55
A-2780	0.30	0.17	0.18	0.21	0.26	0.48
DAOY	0.84	0.46	0.20	0.22	0.30	0.41
Hep3B	0.78	0.62	0.26	0.22	0.25	0.24
SK-OV-3	0.64	0.44	0.21	0.32	0.49	0.51
NCI-H460	0.66	0.56	0.38	0.19	0.19	0.15
Huh-7	0.28	0.15	0.13	0.13	0.11	0.25
U87-MG	1.10	0.44	0.08	0.09	0.11	0.16
ACHN	0.93	0.84	0.61	0.50	0.42	0.51
SNB-19	0.59	0.26	0.21	0.26	0.26	0.46
G361	0.63	0.49	0.51	0.70	0.73	
MCF-7	0.92	0.39	0.28	0.28	0.21	
BT-474	0.87	1.79	1.58	1.72	1.61	1.56
HT-1080	0.24	0.07	0.07	0.06	0.12	0.38
DU145	0.78	0.53	0.57	0.35	0.28	0.36

PC3	0.77	0.42	0.31	0.70	0.24	
HL60	1.59	1.55	1.37	1.34	1.12	0.59

Table S5. IC50 values for BxPC3, A2780, MiaPaca-2, A375 and HCT 116 cells.

**Cell line**	**IC_{50}**
BxPC3	T/C: 0.07/0.42 mg/mL
A2780	T/C: 0.07/0.42 mg/mL
MiaPaca-2	T/C: 0.025/0.15 mg/mL
A375	T/C: 0.035/0.21 mg/mL
HCT 116	T/C: 0.025/0.15 mg/ml
